# Relationship of Plasma Galectin-3 to Renal Function in Patients With Heart Failure: Effects of Clinical Status, Pathophysiology of Heart Failure, and Presence or Absence of Heart Failure

**DOI:** 10.1161/JAHA.112.000760

**Published:** 2012-10-25

**Authors:** Deepa M. Gopal, Maya Kommineni, Nir Ayalon, Christian Koelbl, Rivka Ayalon, Andreia Biolo, Laura M. Dember, Jill Downing, Deborah A. Siwik, Chang-seng Liang, Wilson S. Colucci

**Affiliations:** Cardiovascular Medicine Section, Department of Medicine, Myocardial Biology Unit, Boston University Medical Center, Boston, MA

**Keywords:** biomarkers, galectin-3, heart failure, kidney

## Abstract

**Background:**

Galectin-3 (GAL-3), a β-galactoside–binding protein, is a new clinical biomarker believed to reflect cardiac remodeling/fibrosis in patients with heart failure (HF). Plasma GAL-3 is inversely related to renal function. It is not known whether the relationship between renal function and GAL-3 is influenced by clinical decompensation, type of HF, or the presence or absence of clinical HF.

**Methods and Results:**

Patients were prospectively categorized as having acute decompensated HF or stable HF on the basis of clinical status and as having HF with reduced left ventricular ejection fraction or HF with preserved left ventricular ejection fraction. Plasma GAL-3 was measured by enzyme-linked immunosorbent assay in patients with HF (n=75), control patients without HF (n=32), and control patients without HF with moderate renal insufficiency (n=12). Compared to controls without HF (14±4 ng/mL), GAL-3 was higher in patients with both acute decompensated HF (23±11 ng/mL) and stable HF (22±10 ng/mL) (*P*<0.001 versus controls for both) but did not differ between acute decompensated HF and stable HF (*P*=0.75). Likewise, GAL-3 was elevated in both HF with preserved left ventricular ejection fraction (23±9 ng/mL) and HF with reduced left ventricular ejection fraction (22±11 ng/mL) (*P*<0.001 versus controls for both) but did not differ between HF with preserved ejection fraction and HF with reduced ejection fraction (*P*=0.37). GAL-3 correlated strongly with estimated glomerular filtration rate, both in patients with HF (*r*=−0.75, *P*<0.001) and in patients without HF (*r*=−0.82, *P*<0.001), and this relationship was unaffected by the presence or absence of clinical HF.

**Conclusions:**

Plasma GAL-3 is inversely related to renal function in patients with and without clinical HF. Concentrations of plasma GAL-3 do not seem to depend on the level of compensation or type of HF. Furthermore, the relationship between GAL-3 and renal function seems to be affected little or not at all by the presence or absence of clinical HF.

## Introduction

Galectin-3 (GAL-3), a β-galactoside–binding protein secreted by immune cells, recently was approved by the US Food and Drug Administration as a prognostic aid in patients with heart failure (HF).^1^ Experimentally, GAL-3 is associated with inflammation and tissue fibrosis,^2,3,4,5,6^ and it has been associated with myocardial fibrosis, ventricular remodeling, and left ventricular dysfunction.^7,8^ GAL-3 expression is increased in experimental cardiomyopathy.^8,9,10,11^ Plasma GAL-3 seems to be a prognostic marker of HF outcomes such as death, recurrent HF, and hospitalization for HF.^12,13,14^

Renal function is a recognized determinant of outcomes in HF,^15,16^ and correction for renal impairment has been found to diminish the ability of GAL-3 to predict outcomes in patients with HF in some^12,17^ but not all^13,14,18^ studies. An inverse relationship between GAL-3 and renal function has been observed in patients with HF,^13,18,19^ leading to the suggestion that increased plasma GAL-3 in HF might be due to renal dysfunction, and further, that the ability of GAL-3 to predict outcomes in HF might reflect, at least in part, the consequences of renal impairment. These studies raise questions about the mechanism by which GAL-3 and renal function are related in patients with HF.

Clinical decompensation is associated with impaired renal function,^2008^ as is HF with preserved ejection fraction (HFpEF) versus reduced ejection fraction (HFrEF).^21,22^ Accordingly, a goal of the present study was to determine whether increased GAL-3 in HF is related to either the level of compensation or the underlying pathophysiology (ie, HFpEF versus HFrEF). Studies of GAL-3 in HF to date have not included patients with and without renal dysfunction in the absence of HF. Therefore, a second goal was to determine (1) whether the relationship between GAL-3 and renal function is specific to patients with HF or is present in patients with renal dysfunction in the absence of HF, and (2) whether this relationship is affected by the presence of HF. To address these questions, plasma GAL-3 was measured in patients with HF who were prospectively categorized as having decompensated versus stable HF and as having HFpEF versus HFrEF. To assess the relative roles of HF and renal impairment, plasma GAL-3 also was measured in patients who did not have HF but displayed renal functional impairment comparable to the HF groups.

## Methods

### Study Population

Six groups of patients were prospectively recruited: (1) patients admitted to the hospital for treatment of decompensated HFrEF (n=21), (2) patients admitted to the hospital for treatment of decompensated HFpEF (n=16), (3) ambulatory patients with chronic stable HFrEF (n=20), (4) ambulatory patients with chronic stable HFpEF (n=18), (5) control patients without HF (n=32), and (6) patients without HF but with moderate renal insufficiency due to chronic kidney disease (CKD) (n=12). Patients were assigned to groups on the basis of a review of the medical record and a history and physical examination performed by one of the investigators. The research protocol was approved by the Institutional Review Board at the Boston University Medical Center. Written informed consent was obtained from all participants before enrollment in the study.

Patients with decompensated HF were identified from patients admitted to the hospital for treatment of an episode of HF complicated by volume overload. The diagnosis of decompensated HF was defined clinically as the presence of worsening symptoms of dyspnea, paroxysmal nocturnal dyspnea, or orthopnea in conjunction with clinical signs of circulatory congestion (elevated jugular venous pressure, hepatojugular reflux, hepatomegaly, or peripheral edema). Patients with decompensated HF were enrolled into the study within 24 hours of admission. Patients with chronic stable HF were recruited from the Cardiomyopathy Clinic. These patients had symptoms that had been stable for at least 2 months, had not been hospitalized during the previous 2 months, were not volume overloaded by clinical examination, and did not require a change in diuretic therapy on that visit. HFrEF was defined as a previous diagnosis of HF and echocardiographic demonstration of left ventricular systolic dysfunction with a left ventricular ejection fraction (LVEF) ≤45%, whereas HFpEF was defined as an LVEF ≥50%. Control patients without HF were recruited from the general cardiology ambulatory clinics or inpatient services. None of the control patients without HF had a history of HF or evidence of lung congestion or left ventricular dysfunction at the time of study. An additional 12 patients without HF but with moderate renal insufficiency due to CKD were recruited from an ambulatory nephrology clinic. All urine sediments were bland at the time of enrollment.

Excluded from enrollment were patients with concomitant acute coronary syndromes within the previous 3 months; primary infectious, inflammatory, or rheumatologic processes; hemodynamic instability requiring intravenous vasoactive drugs; aortic stenosis; malignancy; or hepatic dysfunction (cirrhosis or active hepatitis).

### Patient Demographics, Risk Factors, and Clinical Laboratory Variables

Patient demographics were recorded upon enrollment. HF risk factors including smoking history, diabetes, cerebrovascular disease, hypertension, and history of ischemic heart disease were determined via clinical chart review or the use of disease-specific medications (ie, antiglycemics or antihypertensive medications). Estimated glomerular filtration rate (eGFR) was determined by the Chronic Kidney Disease Epidemiology Collaboration formula: eGFR=141×min (Scr/k,1)^a^×max (Scr/k,1)^−1.209^×0.993^Age^×1.018 (if female)× 1.159 (if black).^2009^

### Biomarker Measurements

Blood samples were collected via venipuncture, centrifuged, and then frozen at −70°C until the assays were performed. GAL-3 was analyzed in the plasma with an enzyme-linked immunosorbent assay (BG Medicine, Waltham, MA). N-terminal pro-B-type natriuretic peptide (NT-proBNP) was analyzed in plasma by an enzyme-linked immunosorbent assay (Alpco Diagnostics, Salem, NH). All specimens were processed in duplicate, and the mean intra-assay coefficient of variation was <7%.

### Statistical Analysis

Descriptive statistics are presented as mean±standard deviation for continuous variables and as percentages for categorical variables. Comparisons among all groups for baseline clinical variables were performed with the Kruskal-Wallis test for continuous variables (as these variables exhibited a non-normal distribution with normality testing) and with the Pearson χ^2^ or Fisher exact test for categorical variables. The Tukey multiple-comparisons test was used for testing all other multiple comparisons. Regression analysis with analysis of covariance (ANCOVA) was used to evaluate the relationship between GAL-3 and influential variables. Correlations of GAL-3 with comorbidities and biomarkers were assessed by Spearman rank correlation, with partial correlations used for controlling of potential confounding variables. Backward linear regression was used for the selection of candidate variables, as it permits all the potential predictors to be considered and it allows for the possibility of a set of variables being evaluated for predictive capability together, even if the individual variables might not be the most predictive. A 2-sided *P*<0.05 was accepted as statistically significant. Analyses were performed with SAS 9.2 (SAS Institute Inc, Cary, NC) and graphics generated with SigmaPlot 10.0 (San Jose, CA).

## Results

### Baseline Characteristics

The mean age of all participants was 62±13 years; 61% were male, and 53% were black. The 6 groups did not differ with regard to race, smoking status, hypertension, ischemic heart disease, cerebrovascular disease, diastolic blood pressure, heart rate, or body mass index. As expected, age and systolic blood pressure were higher and female sex was more common in patients with HFpEF than in those with HFrEF. In the CKD group, the predominant origins of renal insufficiency were hypertension (42%) and diabetes (25%) (Table 1).

**Table 1.** Baseline Characteristics (N=119)

		HFrEF		HFpEF		
	Controls (n=32)	Stable (n=20)	Decompensated (n=21)	Stable (n=18)	Decompensated(n=16)	CKD (n=12)
Age,* y	59±11	60±12	61±13	69±10	68±15	58±15
Female,† n (%)	15 (47)	1 (5)	4 (19)	10 (56)	10 (63)	7 (58)
Black race, n (%)	9 (28)	10 (50)	15 (71)	11 (61)	11 (69)	7 (58)
BMI, kg/m^2^	31±5	31±8	31±7	33±8	34±7	29±6
SBP,* mm Hg	126±14	120±26	130±23	136±20	138±19	131±14
DBP, mm Hg	74±7	71±9	77±11	71±10	73±13	74±11
HR,* bpm	74±7	67±11	77±14	68±13	73±12	73±10
LVEF,* %	62±4	25±11	26±12	62±7	61±7	62±6
NYHA class†						
I	—	4 (20)	0 (0)	10 (56)	0 (0)	—
II	—	14 (70)	0 (0)	8 (44)	0 (0)	—
III	—	2 (10)	5 (24)	0	9 (54)	—
IV	—	0 (0)	16 (76)	0	7 (46)	—
Smoker, n (%)	4 (13)	4 (20)	8 (38)	4 (22)	3 (19)	2 (18)
Hypertension, n (%)	22 (69)	16 (80)	17 (81)	16 (90)	15 (94)	8 (67)
Diabetes mellitus, n (%)	10 (31)	13 (65)	11 (52)	10 (56)	12 (75)	6 (50)
Cerebrovascular disease, n (%)	2 (6)	3 (15)	1 (5)	4 (22)	4 (25)	0 (0)
Ischemic heart disease, n (%)	10 (32)	10 (50)	10 (48)	5 (28)	4 (25)	2 (17)
eGFR,* mL/min per 1.73 m^2^	86±25	69±30	68±32	46±23	54±30	33±16
Creatinine,* mg/dL	0.9±0.3	1.7±2.0	1.5±0.8	1.8±0.9	1.6±1.0	2.7 ± 2.2
Plasma NT-proBNP,* fmol/mL	536±382	879±769	3496±2361	1262±602	2269±1337	N/A
Plasma GAL-3,* ng/mL	14±4	21±11	23±12	23±9	22±10	36±11

HFrEF indicates HF with reduced LVEF; HFpEF, HF with preserved LVEF; BMI, body mass index; SBP, systolic blood pressure; DBP, diastolic blood pressure; HR, heart rate; LVEF, left ventricular ejection fraction; NYHA Class, New York Heart Association Functional Class; and eGFR, estimated glomerular filtration rate.

* *P*<0.001 for continuous variables compared across groups by the Kruskal-Wallis test.

† *P*<0.001 for categorical variables compared across groups by χ^2^ test or Fisher exact test.

The mean of the eGFRs in the 4 HF groups (60±30 mL/min per 1.73 m^2^) was lower than in the non-HF control group (86 25 mL/min per 1.73 m^2^, *P*<0.001), as was the eGFR in the CKD group (34±16 mL/min per 1.73 m^2^, *P*<0.001). Plasma NT-proBNP, which was 536±382 fmol/mL in control patients without HF, was moderately elevated in patients with stable HFrEF and HFpEF and markedly elevated in patients with decompensated HFrEF and HFpEF.

### GAL-3 in HF Groups

**Figure 1.**
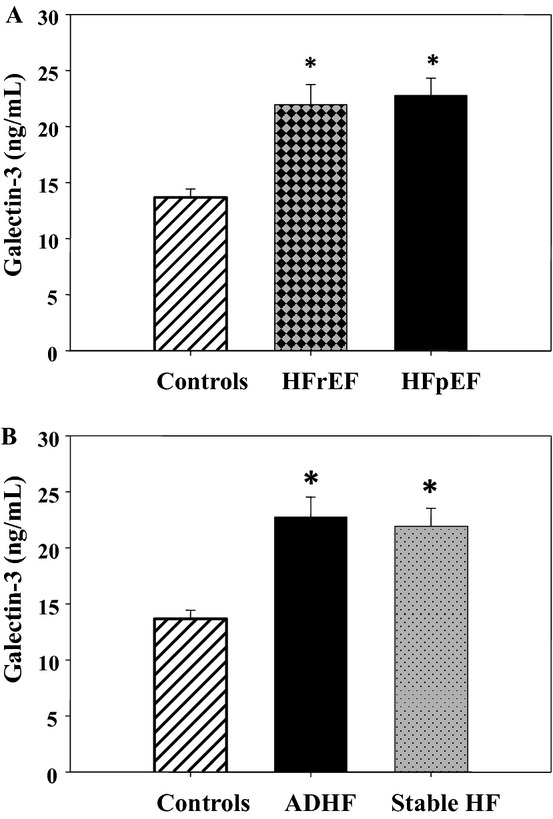
Relationship of plasma GAL-3 levels to the type of HF (A) or level of decompensation (B). A, Plasma GAL-3 in 32 controls without HF, 40 patients with HF with reduced LVEF (HFrEF), and 35 patients with HF with preserved LVEF (HFpEF). GAL-3 was elevated to a similar degree in patients with HFrEF vs HFpEF. *P<0.05 vs control. B, Plasma GAL-3 in 32 controls without HF, 37 patients admitted to the hospital with acute decompensated HF (ADHF), and 38 ambulatory patients with chronic stable HF. GAL-3 was elevated to a similar degree in patients with decompensated vs stable HF. *P<0.05 vs control.

Plasma GAL-3 was 14±4 ng/mL in control patients without HF and was elevated in patients with stable (21±11 ng/mL) and decompensated (23±12 ng/mL) HFrEF and in patients with stable (23±9 ng/mL) and decompensated (22±10 ng/mL) HFpEF (*P*<0.05 versus control patients without HF for all). There were no differences among any of the HF groups (Figure 1). Across all HF groups, plasma GAL-3 did not correlate with age, sex, diabetes, history of coronary artery disease, hypertension, smoking history, systolic blood pressure, diastolic blood pressure, or body mass index. However, in patients with HF, GAL-3 correlated with NT-proBNP (*r*=0.44, *P*<0.001). The relationship between GAL-3 and NT-pro-BNP persisted but was substantially weakened after adjustment for age and eGFR (*r*=0.23, *P*=0.06).

**Figure 2.**
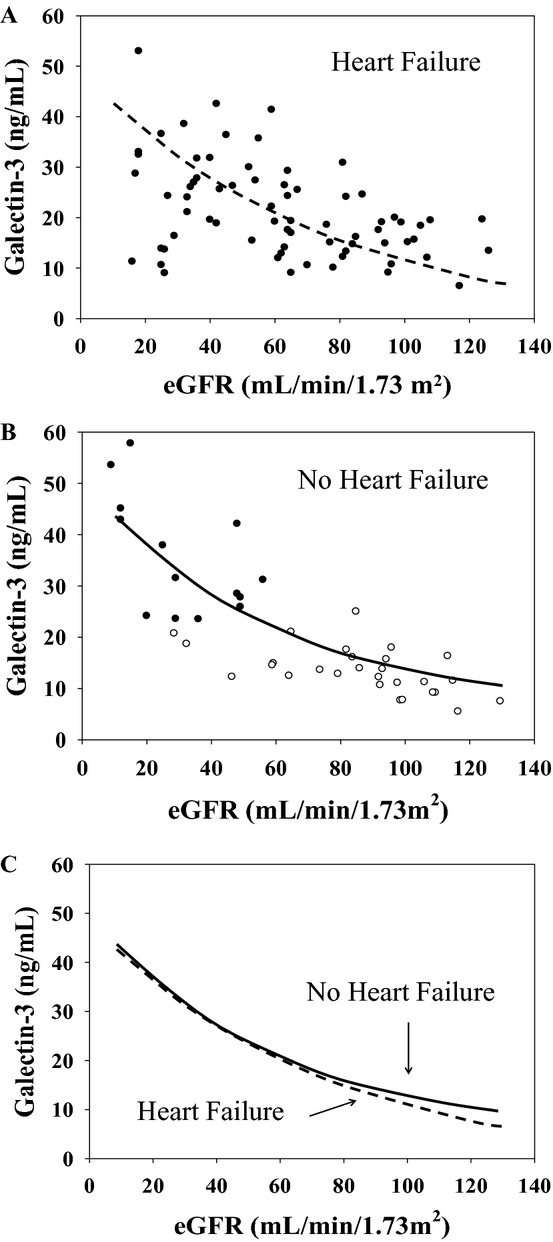
Relationship between plasma GAL-3 and renal function in patients with HF (A) and patients without HF (B). A, Strong curvilinear relationship (*r*=−0.75, *P*<0.001) between plasma GAL-3 and estimated glomerular filtration rate (eGFR) in 75 patients with HF depicted in Figure 1. B, Strong curvilinear relationship (*r*=−0.82, *P*<0.001) between GAL-3 and eGFR in 44 subjects without HF over a range of renal functions comparable to the HF groups. Open circles indicate control subjects; closed circles, patients with CKD. C, Similarity of the relationship between GAL-3 and eGFR in subjects with HF (dashed line) or without HF (solid line) over comparable ranges of renal functions.

In the combined HF groups, GAL-3 was inversely correlated with eGFR (*r*=−0.75, *P*<0.001) (Figure 2A). This relationship remained strong after adjustment for age, LVEF, and NT-proBNP (*r*=−0.64, *P*<0.001).

### Plasma GAL-3 Versus Type of HF and Compensation State

GAL-3 was elevated in patients with both HFpEF and HFrEF compared to control patients without HF but did not differ between HFpEF and HFrEF (*P*=0.37) (Figure 1A). Likewise, plasma GAL-3 did not differ between patients with decompensated versus stable HF (*P*=0.75) (Figure 1B).

### Relationship to Renal Function

Mean GAL-3 in the CKD group (36±11 ng/mL) was higher than in the non-HF control group (14±4 ng/mL) or the HF groups (combined mean=22±10 ng/mL). When data from all 6 groups (non-HF controls, HF, CKD) were pooled, a striking inverse curvilinear relationship was apparent for GAL-3 versus eGFR (*r*=−0.77, *P*<0.001). This relationship was significant both in the patients with HF (*r*=−0.75, *P*<0.001) (Figure 2A) and in the patients without HF (ie, the combined controls without HF and the patients without HF but with impaired renal function) (*r*=−0.82, *P*<0.001) (Figure 2B). The relationships between GAL-3 and eGFR were identical in the HF and non-HF groups (Figure 2C). By linear regression analysis of the relationship between eGFR and GAL-3, the β-coefficient of eGFR is −0.025 (95% confidence interval, −0.022 to −0.023, *P*<0.001, per standard deviation of log GAL-3) with R^2^=0.60. When HF status is added to the regression, the β-coefficient of eGFR is unchanged (−0.025; R^2^=0.60). Backward linear regression of log GAL-3 in HF patients was performed with candidate predictors of age, LVEF, sex, and eGFR. With a significance level of 0.05 for variable retention in the model, only eGFR remained predictive of GAL-3 (eGFR: β-coefficient −0.022; 95% CI, −0.020 to −0.024; *P*<0.001, per standard deviation of log GAL-3).

## Discussion

The major findings of this study are that (1) the inverse relationship between plasma GAL-3 and renal function is independent of the level of clinical decompensation or the pathophysiology of HF, and (2) the presence or absence of clinical HF has little or no effect on the relationship between GAL-3 and renal function.

### Relationship to Clinical Compensation

Brain natriuretic peptides, including NT-proBNP, are the most frequently used clinical biomarkers in patients with HF, in whom the elevation in plasma brain natriuretic peptides is strongly related to the severity of clinical decompensation.^24,25^ This relationship is well illustrated in the present study, in which patients were prospectively categorized according to clinical status. In patients with chronic stable HF, NT-proBNP was only modestly (2- to 3-fold) elevated compared to control patients without HF, whereas in decompensated patients, NT-proBNP was markedly (5- to 7-fold) elevated, which thus confirms the prospective clinical categorization used in this study.

Prior studies have not examined the relationship between plasma GAL-3 and the severity of decompensation in HFrEF and HFpEF in a prospective manner. Recently, we observed that episodes of decompensation are associated with increased plasma levels of 3 biomarkers related to the interstitial matrix, possibly reflecting an increase in matrix turnover.^26^ Because GAL-3 is associated with interstitial fibrosis,^7,8^ it seemed likely that plasma GAL-3 might also increase transiently with an episode of decompensation. Contrary to our thesis, GAL-3 was not affected by episodes of decompensation, regardless of the type of HF (HFrEF or HFpEF).

### Relationship to HF Pathophysiology

Prior studies of plasma GAL-3 in HF have focused primarily on patients with HFrEF^13,18^ or on mixed populations of patients with HFrEF and HFpEF.^12,14,19^ No prior study has systematically compared plasma GAL-3 levels between HFpEF and HFrEF in patients with both stable and decompensated HF. Animal studies suggest that GAL-3 is involved in the fibrotic process, and limited clinical data support the notion that plasma GAL-3 is reflective of myocardial fibrosis.^27^ Because HFpEF is associated with interstitial fibrosis^28^ and increased plasma levels of biomarkers of matrix turnover,^29,30^ it seemed likely that GAL-3 would be more elevated in patients with HFpEF than in those with HFrEF. However, GAL-3 levels were not different in patients with HFrEF versus HFpEF. As discussed previously, GAL-3 was not affected by the level of compensation, and likewise, GAL-3 levels in patients with HFrEF and HFpEF were similar regardless of the level of compensation. Thus, although plasma GAL-3 is elevated in patients with HF, the degree of elevation is not related to the severity of decompensation or the underlying type of HF.

### Relationship to Renal Function

Plasma GAL-3 correlated strongly (*r*=−0.75, *P*<0.001) with eGFR across all of the HF groups, regardless of type or severity of HF (see Figure 2A). Furthermore, this relationship remained strong after adjustment for age, LVEF, and NT-proBNP. In further support of the importance of renal function in predicting GAL-3, eGFR was the strongest predictor of GAL-3 levels in patients with HF when evaluated with other predictors, such as age, LVEF, and sex in backwards linear regression.

To further characterize the relationship of GAL-3 with renal function in the absence of HF, we measured GAL-3 in a group of patients with moderate renal insufficiency due to CKD but no HF. As with the patients who had HF, GAL-3 correlated strongly (*r*=−0.82, *P*<0.001) with eGFR in patients without HF across a wide range of eGFRs that was comparable to the range in the HF groups. Our data thus indicate that renal function is a major determinant of GAL-3 levels, both in patients with HF and in patients without HF. Of note, the curvilinear relationships between GAL-3 and eGFR were superimposable for patients with and without HF (see Figure 2C), which suggests that renal impairment, rather than HF, could be the major determinant of GAL-3 in patients with HF.

There are several potential mechanisms for increased GAL-3 levels in the setting of renal impairment. First, it is possible that GAL-3 (molecular weight ≍30 kDa) clearance is via the kidney and thus serves primarily as a marker of reduced renal function. Second, there could be increased production of GAL-3 by the kidney.^31,32^ Third, it is possible that GAL-3 is produced in the kidney and exerts profibrotic effects resulting in renal function impairment. However, evidence also exists that GAL-3 could protect the kidney from ischemia/reperfusion^32,33^ and in CKD.^34^ Finally, it is possible that increased GAL-3 in HF reflects production by organs other than the heart and kidneys, particularly in the setting of systemic inflammation^35,36,37^ that can occur in both HF and CKD.^38^

### Limitations

Certain limitations of this study should be considered. First, the relatively small number of patients might have reduced the ability to distinguish differences between patient groups with stable versus decompensated HF or HFrEF versus HFpEF. A second limitation is the difficulty of controlling for all factors that might affect GAL-3 level. Although it is possible that these limitations might have led to underestimation of the differences between groups, we think it is unlikely that important differences were missed, as the data were sufficiently robust to detect the striking effect of renal function on plasma GAL-3 in patients with or without HF. Finally, because of the sample sizes, it was not possible to perform a formal interaction test for the effect of HF on the relationship of GAL-3 to eGFR. Nevertheless, as Figure 2C illustrates, the influence of HF, if any, seems to be small.

### Summary

GAL-3 recently was introduced as a biomarker for the assessment of prognosis in patients with HF.^1^ Here we demonstrate that renal impairment is a major determinant of plasma GAL-3 in patients with or without HF. Concentrations of plasma GAL-3 do not seem to depend on the level of compensation or type of HF. Furthermore, the relationship between GAL-3 and renal function seems to be affected little or not at all by the presence or absence of clinical HF. These observations raise important considerations with regard to both the use of GAL-3 in clinical practice and the interpretation of GAL-3 values in outcome trials in patients with HF.

## References

[1] US Food and Drug Administration Device market approval under the federal food, drug, and cosmetic act. http://www.accessdata.fda.gov/cdrh_docs/pdf9/K093758.pdf.

[2] Christenson RH Duh SH Wu AH Smith A Abel G deFilippi CR Wang S Adourian A Adiletto C Gardiner P Multi-center determination of galectin-3 assay performance characteristics: anatomy of a novel assay for use in heart failure. Clin Biochem. 2010;43:683-690. 20153309 10.1016/j.clinbiochem.2010.02.001

[3] Henderson NC Mackinnon AC Farnworth SL Kipari T Haslett C Iredale JP Liu FT Hughes J Sethi T Galectin-3 expression and secretion links macrophages to the promotion of renal fibrosis. Am J Pathol. 2008;172:288-298. 18202187 10.2353/ajpath.2008.070726 PMC2312353

[4] Henderson NC Sethi T The regulation of inflammation by galectin-3. Immunol Rev. 2009;230:160-171. 19594635 10.1111/j.1600-065X.2009.00794.x

[5] Krzeslak A Lipinska A Galectin-3 as a multifunctional protein. Cell Mol Biol Lett. 2004;9:305-328. 15213811

[6] Ochieng J Furtak V Lukyanov P Extracellular functions of galectin-3. Glycoconj J. 2004;19:527-535. 14758076 10.1023/B:GLYC.0000014082.99675.2f

[7] Liu YH D'Ambrosio M Liao TD Peng H Rhaleb NE Sharma U Andre S Gabius HJ Carretero OA N-acetyl-seryl-aspartyl-lysyl-proline prevents cardiac remodeling and dysfunction induced by galectin-3, a mammalian adhesion/growth-regulatory lectin. Am J Physiol Heart Circ Physiol. 2009;296:H404-H412. 19098114 10.1152/ajpheart.00747.2008 PMC2643891

[8] Sharma UC Pokharel S van Brakel TJ van Berlo JH Cleutjens JP Schroen B Andre S Crijns HJ Gabius HJ Maessen J Pinto YM Galectin-3 marks activated macrophages in failure-prone hypertrophied hearts and contributes to cardiac dysfunction. Circulation. 2004;110:3121-3128. 15520318 10.1161/01.CIR.0000147181.65298.4D

[9] Schroen B Heymans S Sharma U Blankesteijn WM Pokharel S Cleutjens JP Porter JG Evelo CT Duisters R van Leeuwen RE Janssen BJ Debets JJ Smits JF Daemen MJ Crijns HJ Bornstein P Pinto YM Thrombospondin-2 is essential for myocardial matrix integrity: increased expression identifies failure-prone cardiac hypertrophy. Circ Res. 2004;95:515-522. 15284191 10.1161/01.RES.0000141019.20332.3e

[10] Sharma UC Pokharel S Evelo CT Maessen JG A systematic review of large scale and heterogeneous gene array data in heart failure. J Mol Cell Cardiol. 2005;38:425-432. 15733902 10.1016/j.yjmcc.2004.12.016

[11] Thandavarayan RA Watanabe K Ma M Veeraveedu PT Gurusamy N Palaniyandi SS Zhang S Muslin AJ Kodama M Aizawa Y 14-3-3 Protein regulates ask1 signaling and protects against diabetic cardiomyopathy. Biochem Pharmacol. 2008;75:1797-1806. 18342293 10.1016/j.bcp.2008.02.003

[12] de Boer RA Lok DJ Jaarsma T van der Meer P Voors AA Hillege HL van Veldhuisen DJ Predictive value of plasma galectin-3 levels in heart failure with reduced and preserved ejection fraction. Ann Med. 2010;43:60-68. 21189092 10.3109/07853890.2010.538080 PMC3028573

[13] Tang WH Shrestha K Shao Z Borowski AG Troughton RW Thomas JD Klein AL Usefulness of plasma galectin-3 levels in systolic heart failure to predict renal insufficiency and survival. Am J Cardiol. 2011;108:385-390. 21600537 10.1016/j.amjcard.2011.03.056 PMC3137764

[14] van Kimmenade RR Januzzi JL Jr Ellinor PT Sharma UC Bakker JA Low AF Martinez A Crijns HJ MacRae CA Menheere PP Pinto YM Utility of amino-terminal pro-brain natriuretic peptide, galectin-3, and apelin for the evaluation of patients with acute heart failure. J Am Coll Cardiol. 2006;48:1217-1224. 16979009 10.1016/j.jacc.2006.03.061

[15] Damman K Navis G Voors AA Asselbergs FW Smilde TD Cleland JG van Veldhuisen DJ Hillege HL Worsening renal function and prognosis in heart failure: systematic review and meta-analysis. J Card Fail. 2007;13:599-608. 17923350 10.1016/j.cardfail.2007.04.008

[16] Smith GL Lichtman JH Bracken MB Shlipak MG Phillips CO DiCapua P Krumholz HM Renal impairment and outcomes in heart failure: systematic review and meta-analysis. J Am Coll Cardiol. 2006;47:1987-1996. 16697315 10.1016/j.jacc.2005.11.084

[17] Lainscak M Coletta AP Sherwi N Cleland JG Clinical trials update from the Heart Failure Society of America Meeting 2009: FAST, IMPROVE-HF, COACH Galectin-3 Substudy, HF-ACTION Nuclear Substudy, DAD-HF, and MARVEL-1. Eur J Heart Fail. 2010;12:193-196. 20042425 10.1093/eurjhf/hfp185

[18] Lok DJ van der Meer P de la Porte PW Lipsic E Van Wijngaarden J Hillege HL van Veldhuisen DJ Prognostic value of galectin-3, a novel marker of fibrosis, in patients with chronic heart failure: data from the DEAL-HF study. Clin Res Cardiol. 2010;99:323-328. 20130888 10.1007/s00392-010-0125-y PMC2858799

[19] Shah RV Chen-Tournoux AA Picard MH van Kimmenade RR Januzzi JL Galectin-3, cardiac structure and function, and long-term mortality in patients with acutely decompensated heart failure. Eur J Heart Fail. 2010;12:826-832. 20525986 10.1093/eurjhf/hfq091 PMC2913048

[20] Liang KV Williams AW Greene EL Redfield MM Acute decompensated heart failure and the cardiorenal syndrome. Crit Care Med. 2008;36:S75-S88. 18158481 10.1097/01.CCM.0000296270.41256.5C

[21] Hillege HL Girbes AR de Kam PJ Boomsma F de Zeeuw D Charlesworth A Hampton JR van Veldhuisen DJ Renal function, neurohormonal activation, and survival in patients with chronic heart failure. Circulation. 2000;102:203-210. 10889132 10.1161/01.cir.102.2.203

[22] Miyagishima K Hiramitsu S Kimura H Mori K Ueda T Kato S Kato Y Ishikawa S Iwase M Morimoto S Hishida H Ozaki Y Long term prognosis of chronic heart failure: reduced vs preserved left ventricular ejection fraction. Circ J. 2009;73:92-99. 19043227 10.1253/circj.cj-07-1016

[23] Levey AS Stevens LA Schmid CH Zhang YL Castro AF III Kusek JW Eggers P Van Lente F Greene T Coresh J for the CKD-EPI (Chronic Kidney Disease Epidemiology Collaboration) A new equation to estimate glomerular filtration rate. Ann Intern Med. 2009;150:604-612. 19414839 10.7326/0003-4819-150-9-200905050-00006 PMC2763564

[24] Cheng V Kazanagra R Garcia A Lenert L Krishnaswamy P Gardetto N Clopton P Maisel A A rapid bedside test for B-type peptide predicts treatment outcomes in patients admitted for decompensated heart failure: a pilot study. J Am Coll Cardiol. 2001;37:386-391. 11216951 10.1016/s0735-1097(00)01157-8

[25] Guglin M Hourani R Pitta S Factors determining extreme brain natriuretic peptide elevation. Congest Heart Fail. 2007;13:136-141. 17541308 10.1111/j.1527-5299.2007.06478.x

[26] Biolo A Fisch M Balog J Chao T Schulze PC Ooi H Siwik D Colucci WS Episodes of acute heart failure syndrome are associated with increased levels of troponin and extracellular matrix markers. Circ Heart Fail. 2010;3:44-50. 19850700 10.1161/CIRCHEARTFAILURE.108.844324

[27] Lin YH Lin LY Wu YW Chien KL Lee CM Hsu RB Chao CL Wang SS Hsein YC Liao LC Ho YL Chen MF The relationship between serum galectin-3 and serum markers of cardiac extracellular matrix turnover in heart failure patients. Clin Chim Acta. 2009;409:96-99. 19747906 10.1016/j.cca.2009.09.001

[28] Zile MR Brutsaert DL New concepts in diastolic dysfunction and diastolic heart failure, Part II: causal mechanisms and treatment. Circulation. 2002;105:1503-1508. 11914262 10.1161/hc1202.105290

[29] Martos R Baugh J Ledwidge M O'Loughlin C Conlon C Patle A Donnelly SC McDonald K Diastolic heart failure: evidence of increased myocardial collagen turnover linked to diastolic dysfunction. Circulation. 2007;115:888-895. 17283265 10.1161/CIRCULATIONAHA.106.638569

[30] Weber KT Sun Y Tyagi SC Cleutjens JP Collagen network of the myocardium: function, structural remodeling and regulatory mechanisms. J Mol Cell Cardiol. 1994;26:279-292. 8028011 10.1006/jmcc.1994.1036

[31] Fernandes Bertocchi AP Campanhole G Wang PH Goncalves GM Damiao MJ Cenedeze MA Beraldo FC de Paula Antunes Teixeira V Dos Reis MA Mazzali M Pacheco-Silva A Camara NO A role for galectin-3 in renal tissue damage triggered by ischemia and reperfusion injury. Transpl Int. 2008;21:999-1007. 18657091 10.1111/j.1432-2277.2008.00705.x

[32] Nishiyama J Kobayashi S Ishida A Nakabayashi I Tajima O Miura S Katayama M Nogami H Up-regulation of galectin-3 in acute renal failure of the rat. Am J Pathol. 2000;157:815-823. 10980121 10.1016/S0002-9440(10)64595-6 PMC1885699

[33] Vansthertem D Cludts S Nonclercq D Gossiaux A Saussez S Legrand A Gabius HJ Toubeau G Immunohistochemical localization of galectins-1 and -3 and monitoring of tissue galectin-binding sites during tubular regeneration after renal ischemia reperfusion in the rat. Histol Histopathol. 2010;25:1417-1429. 20865664 10.14670/HH-25.1417

[34] Okamura DM Pasichnyk K Lopez-Guisa JM Collins S Hsu DK Liu FT Eddy AA Galectin-3 preserves renal tubules and modulates extracellular matrix remodeling in progressive fibrosis. Am J Physiol Renal Physiol. 2010;300:F245-F253. 20962111 10.1152/ajprenal.00326.2010 PMC3023228

[35] Dancer JY Truong LD Zhai Q Shen SS Expression of galectin-3 in renal neoplasms: a diagnostic, possible prognostic marker. Arch Pathol Lab Med. 2010;134:90-94. 20073610 10.5858/2008-0392-OAR1.1

[36] de Oliveira JT de Matos AJ Gomes J Vilanova M Hespanhol V Manninen A Rutteman G Chammas R Gartner F Bernardes ES Coordinated expression of galectin-3 and galectin-3-binding sites in malignant mammary tumors: implications for tumor metastasis. Glycobiology. 2010;20:1341-1352. 20591828 10.1093/glycob/cwq103

[37] Kang EH Moon KC Lee EY Lee YJ Lee EB Ahn C Song YW Renal expression of galectin-3 in systemic lupus erythematosus patients with nephritis. Lupus. 2009;18:22-28. 19074165 10.1177/0961203308094361

[38] Mann DL Inflammatory mediators in heart failure: homogeneity through heterogeneity. Lancet. 1999;353:1812-1813. 10359399 10.1016/S0140-6736(99)90069-7

