# Correction

**DOI:** 10.1161/JAHA.113.000194

**Published:** 2013-06-21

## Introduction

In the article by Gopal et al, “Relationship of Plasma Galectin‐3 to Renal Function in Patients With Heart Failure: Effects of Clinical Status, Pathophysiology of Heart Failure, and Presence or Absence of Heart Failure,” which was published online on September 6, 2012, and appears with the October 2012 issue (*J Am Heart Assoc*. 2012;1:e000760 doi: 10.1161/JAHA.112.000760), a correction was needed.

During the revision process, a transcription error led to several incorrect data points in Figure 2A as it was originally published (included with this erratum for reference). The corrected figure (included with the current online version of the article) shows the relationship between galectin‐3 (GAL‐3) and estimated glomerular filtration rate (eGFR) in patients with heart failure (HF) (Figure 2A) or without HF (Figure 2B). In Figure 2B, open circles depict control patients, and closed circles depict patients with chronic kidney disease.

This error was entirely graphical in nature and had no effect on any aspect of the data presented, statistical analyses, or conclusions. In particular, a strong relationship was seen between GAL‐3 and eGFR in patients with HF (correlation coefficient, *r*=−0.75, *P*<0.001) (Figure 2A) and in patients without HF (correlation coefficient, *r*=−0.82, *P*<0.001). A formal evaluation of the impact of HF status (HF or no HF) on the relationship between GAL‐3 and eGFR was conducted by statistical interaction testing (SAS) using a regression model with GAL‐3 as the dependent variable. There was no statistical interaction of HF (*P*=0.12), thus supporting our conclusion that the presence of HF did not affect the relationship between GAL‐3 and eGFR. For depiction purposes only, best‐fit lines were generated for the data in Figure 2 using a polynomial inverse third‐order regression model (Y=Yo+a/x+b/x^2^+c/x^3^) which exhibited a good fit for both HF (R^2^=0.61) and non‐HF (R^2^=0.72) subjects.


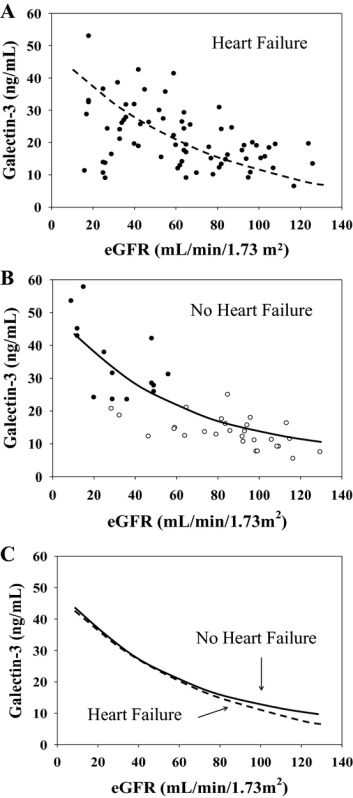


The corrections have been made to the current online version of the article, which is available at http://jaha.ahajournals.org/content/1/5/e000760.full. The authors regret the errors.

